# Self-reported experience of orofacial injury, preventive practice, and knowledge of Iranian adolescent martial art athletes towards sports-related orofacial injuries

**DOI:** 10.1186/s13102-021-00363-4

**Published:** 2021-10-26

**Authors:** Ali Esmaeilpoor, Simin Z. Mohebbi, Navid Moghadam, Mina Ahmadian, Samaneh Razeghi, Mohammad R. Khami

**Affiliations:** 1 School of Dentistry, Tehran University of Medical Sciences, Tehran, Iran; 2 Research Center for Caries Prevention, Dentistry Research Institute, Tehran University of Medical Sciences, Tehran, Iran; 3 Department of Community Oral Health, School of Dentistry, Tehran University of Medical Sciences, Tehran, Iran; 4 Sports Medicine Research Center, Neuroscience Institute, Tehran University of Medical Sciences, Tehran, Iran; 5 Iran Sports Medicine Federation, Tehran, Iran; 6 Department of Pediatric Dentistry, Faculty of Dentistry, Ilam University of Medical Sciences, Ilam, Iran

**Keywords:** Contact sports, Orofacial injuries, Adolescent

## Abstract

**Background:**

Increased engagement of adolescents in martial arts exposes them to a relatively high risk of experiencing orofacial injuries. We evaluated self-reported experience of orofacial injuries, preventive practice, and knowledge of management of sport-related orofacial injuries and related factors in adolescent Karate and Taekwondo athletes in Iran.

**Methods:**

This cross-sectional study was conducted on Iranian martial arts athletes including Karate and Taekwondo aged 11–17-year-old in 2020 via an online questionnaire. The anonymous self-administrated questionnaire had four parts: background (age, gender, duration of sports activity, training sessions per week, and previous education on orofacial injury); self-reported experience of orofacial injury; preventive practice including mouthguard and helmet use; and knowledge of emergency management of orofacial injuries. Preventive practice and knowledge scores were calculated by summing up the scores of corresponding questions (possibly range 0–7). A linear regression model and the Pearson correlation served for statistical analysis.

**Results:**

Totally, 295 athletes with a mean age of 14.56 ± 1.91 years participated in the study. A quarter (n = 74, 25.1%) of athletes stated that they had received training on prevention of orofacial injuries. Only 3.7% (n = 11) of the participants reported the use of custom-made mouthguards, and 62% (n = 183) reported a positive history of orofacial injuries. A significant relationship existed between self-reported orofacial injury and age (β = − 0.32, *p* < 0.001), hours of training per week (β = 0.12, *p* = 0.037), type of sport (β = 0.11, *p* = 0.049), and previous training on orofacial injuries (β = − 0.14, *p* = 0.010). No difference existed in the history of orofacial injury between females (n = 114) and males (n = 69) (*p* = 0.374). The mean score of self-reported preventive practice and knowledge of management of orofacial injuries was 3.53 ± 1.82 (out of 7) and 1.67 ± 1.10 (out of 7), respectively. Age (β = 0.19, *p* = 0.002) and history of orofacial injury (β = − 0.15, *p* = 0.010) were associated with the score of self-reported preventive practice. Female athletes (β = 0.11, *p* = 0.048) and athletes who exercised more per week (β = 0.15, *p* = 0.012) had significantly more knowledge on management of orofacial injuries.

**Conclusion:**

Adolescent athletes had relatively undesirable preventive practices and a distinct lack of knowledge. The high occurrence of self-reported orofacial injuries indicates the importance of more education and stricter rules for the athlete population.

## Background

Orofacial injuries are a significant public health problem due to their relatively high frequency in the society and the extensive impact they may have on a person’s quality of life [1, 2]. Children and adolescents are a high-risk group for orofacial injuries [3]. In a meta-analysis by Petti et al*.* [1], nearly one in five children aged 11–13 years had a history of traumatic dental injuries (TDIs); moreover, males were more likely to experience traumatic dental injuries (TDIs) than females [1]. Based on the results of the latest national oral health survey in Iran, about 5% of the 12-year-old and 6% of the 15-year-old Iranians experienced TDIs, which were more prevalent in boys in both age groups [4].

Increased participation in sports activities comes with a significant risk of sustaining orofacial injuries. On the other hand, the popularity of martial arts in combat form in both genders and the engagement of children and young adults in such sports expose them to a relatively high risk of experiencing orofacial injuries at an early age [5–8]. Fortunately, most sports-related orofacial injuries are preventable through using equipment such as mouthguards, facemasks and helmets [9]. In a systematic review, Knapik et al*.* found that using mouthguards during sports activities reduced the risk of orofacial injuries [10]. Furthermore, the American Dental Association and the Academy for Sports Dentistry have recommended the use of custom-fitted mouthguards in all activities with a risk of orofacial injuries [5, 9, 11, 12].

American Academy of Pediatric Dentistry has stated that falls, collisions, contact with hard surfaces, and contact from sports-related equipment poses an associated risk of orofacial injuries with all sporting activities [9]. Martial arts include a variety of formal traditions selected by athletes for self-defense, competition, physical fitness, motor development, and emotional growth [13, 14]. There are both noncombat forms with less risk of injury, and combat forms with increased potential for injury [13]. Taekwondo and Karate are two popular disciplines of martial arts among Iranian adolescents [15, 16]. Iranian children and adolescents of both genders in different age groups regularly practice these two martial arts, which belong to the group of contact sports and may lead to injury to the athletes not only during competitions but also in training sessions [15, 16].

Some domestic studies have evaluated orofacial injuries in martial arts and showed that the prevalence of head and neck injury was relatively high in comparison with other types of injuries. For instance, Ziaee et al*.* found that in Karate, 61% of all injuries were located in the head and neck area in athletes younger than 30 years old [16]. In another study by Halabchi et al*.,* 55.4% of all injuries were located in the head, neck and face in female Karate athletes [7]. Moreover, Ziaee et al*.* reported that head and neck injuries accounted for about 7.5% of all injuries in male black-belt taekwondo competitors aged 17–32 years old [15].

Considering the sparse data on the knowledge of orofacial injury prevention and mouthguard use in Iranian adolescent martial arts athletes and to establish effective educational strategies for orofacial injuries in this high-risk group, the present study was conducted to evaluate self-reported experience of orofacial injuries and related factors, self-reported preventative practice, and knowledge of the management of sports-related orofacial injuries in 11–17 year-old Karate and Taekwondo athletes in Iran in 2020.

## Materials and methods

### Sampling and data collection

This descriptive-analytical cross-sectional study was conducted on 11–17-year-old Karate and Taekwondo athletes in Iran in 2020. This age group was selected since there is a general notion that adolescents experience accidental injuries during contact sports [17]; moreover, it has been shown that a small proportion of young athletes in diverse sport activities are aware of the importance of using mouthguards among them only 3% actually used them [18]. Additionally, increased participation of females in sports and their similar exposure to the risk factors compared with males [19, 20] were the reasons for recruitment of adolescent athletes of both genders. Among different martial arts, Karate and Taekwondo have the highest number of athletes in both genders in Iran.

Based on a previous study indicating a prevalence of 61% for head and neck injuries [16] and considering α = 0.05 and β = 0.8, the minimum sample size was estimated at 253.

All contact sports clubs were closed in Iran at the time of data collection due to the COVID-19 pandemic. Therefore, the athletes were surveyed via an online questionnaire prepared in Google Docs (Google Inc., Googleplex, Mountain View, California, USA). After coordination with Taekwondo and Karate federations, the Public Relations of both federations published the link of the online questionnaire in their social networks (WhatsApp Inc., Mountain View, California, USA). This link was provided to athletes on a daily basis for a week from 8 to 15th September 2020. Two reminders were sent in the social networks during this period. Participation in the study was voluntary. All participants were informed about the objectives of the study; therefore, those who were willing to participate in the study filled out the questionnaire.

The inclusion criteria were (1) male or female gender, (2) age 11 and 17 years old, and (3) being a member of the official social network of Taekwondo or Karate Federation. Gender and age of participants were self-reported through the self-administrated questionnaire; and access to members of the official social network of Taekwondo or Karate Federation was through the Public Relations of both federations. The only exclusion criterion was reluctance to participate in the study.

### Questionnaire

An anonymous self-administrated questionnaire was adopted from previous studies [11, 21, 22]. After collecting the questions, a panel of experts including two community oral health experts, one pediatric dentist, one sport medicine specialist, and one epidemiologist assessed the content validity of the questionnaire. The reliability of the questionnaire was assessed through a test–retest approach on 10 Taekwondo athletes at an interval of 10 days. These athletes were excluded from the main study. The Kappa coefficient ranged from 0.71 to 0.93 for different questions which means a good test–retest reliability [23].

The questionnaire was in Farsi and comprised four parts as follows:

- Background information (age, gender, years of practice, number and duration of training sessions per week, and previous training on prevention and management of orofacial injury);
- Self-reported experience of orofacial injury: a “yes/no” question
- Self-reported preventive practice: five questions on the use of protective gear (mouthguard and helmet) using multiple-choice as well as “yes, no, and I do not know” answers. Unfavorable and “I do not know” answers were scored 0 and favorable answers were scored 1. One question on the type of athlete mouthguard had weighted scores based on the answers (custom-made = 3, mouth-formed = 2, stock = 1, not using = 0). The self-reported preventive practice score of each athlete was calculated through summing up the scores of five questions (range: 0–7) [24, 25]. A multiple-choice question addressed the reasons for not using the mouthguard.
- Knowledge of emergency management of orofacial injuries: seven questions with multiple-choice and “yes, no, and I do not know” answers. A score of 0 was given to false or “I do not know” answers and a score of 1 was assigned to correct answers. The knowledge score of each athlete was calculated by summing up the scores of the seven questions (range: 0–7) [24, 25].

Based on achievable range of scores, the level of both self-reported preventive practice, and knowledge of respondents were categorized as inadequate (0–3), moderate (> 3 and < 5), and adequate (> 5).

### Ethics

Ethical approval was obtained from the Ethics Committee of Tehran University of Medical Sciences (IR.TUMS.DENTISTRY.REC.1399.016). All steps of the study were performed in accordance with the Declarations of Helsinki. The study was voluntary and the responses were anonymous. All the participants were informed about the objectives of the study. Informed consent was obtained from participants and their parents via a link sent to the parent’s cellphone.

### Statistical analysis

After collecting the questionnaires, the answers were scored and the results were analyzed using descriptive statistics, Backward linear regression model, and Pearson correlation coefficient. Backward linear regression model was used to determine the effects of selected independent variables on athletes’ self-reported experience of orofacial injuries, self-reported preventative practice, and knowledge of the management of sports-related orofacial injury, as multivariate analysis. The association between self-reported practice and knowledge was explored using the Pearson correlation coefficient. P-values less than 0.05 were considered statistically significant. Data analysis was performed using SPSS version 25 (Chicago, IL, USA).

## Results

In total, 295 questionnaires were collected. The mean age of the participants was 14.56 ± 1.91 years old. Totally, 59.0% of the participants (n = 147) were female, and 78.0% (n = 230) did Taekwondo. The average duration of practicing these sports was 5.02 ± 2.77 years, and 230 (78.0%) athletes had more than three years of experience. Of all the participants 56.6% (n = 167) trained more than 5 h per week. Of all the participants who completed the questionnaires, 74 (25.1%) stated that they had received training on prevention of orofacial injuries. Table 1 shows the characteristics of the study participants based on the sport.

**Table 1:** Characteristics of the study participants (n = 295)

	Karate n (%)	Taekwondo n (%)	Total n (%)
*Gender*			
Male	24 (36.9)	97 (42.2)	121 (41.0)
Female	41 (63.1)	133 (57.8)	174 (59.0)
*Number of years of sport experience*			
< 3 years	13 (4.4)	52 (17.6)	65 (22.0)
≥ 3 years	51 (17.3)	179 (60.7)	230 (78.0)
*Training sessions per week*			
1–2 h/week	9 (13.8)	25 (10.9)	34 (11.5)
3–5 h/week	10 (15.4)	84 (36.5)	94 (31.9)
≥ 5 h/week	46 (70.8)	121 (52.6)	167 (56.6)
*Previous training on prevention and management of orofacial injuries*			
Yes	28 (43.1)	46 (20.0)	74 (25.1)
No	37 (56.9)	184 (80.0)	221 (74.9)

One hundred and eighty-three subjects (62%) reported a positive history of orofacial injuries. There was, however, no significant difference in the history of orofacial injuries between genders (females: n = 114, 65.5%, males: n = 69, 57%) (*p* = 0.37). A significant relationship was found between self-reported experience of orofacial injury and age (β = − 0.32, *p* < 0.001), hours of training per week (β = 0.12, *p* = 0.037), and previous training on prevention and management of orofacial injuries (β = − 0.14, *p* = 0.010). Taekwondo players reported significantly more self-reported experience of orofacial injuries compared to Karate players (β = 0.11, *p* = 0.049) (Table 2).

**Table 2:** Results of linear regression model controlling for demographics on factor associated with athletes’ self-reported experience of orofacial injuries (n = 295)

	Unstandardized coefficients	Standardized coefficients^a^	*p* value
*Self-reported experience of orofacial injuries*			
Age	− 0.8	− 0.3	< 0.001
Type of sport	0.1	0.1	0.049
Training sessions per week	0.1	0.1	0.037
Previous training on orofacial injuries	− 0.2	− 0.1	0.010

^a^Scale-standardized

**Fig. 1:**
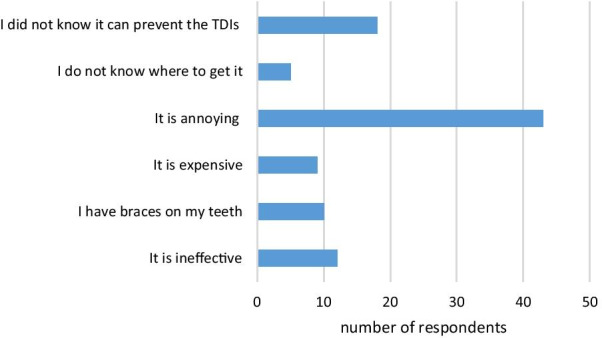
Reasons for not using the mouthguard stated by athletes (n = 97)

Of all participants, 71.5% (n = 211) indicated that they usually used a mouthguard during practice or a match. The most common types of mouthguards used by athletes were stocks (n = 117, 39.7%) and mouth-formed mouthguards (n = 90, 30.5%). Only 3.7% (n = 11) of the participants reported the use of custom-made mouthguards. Table 3 shows more details about mouthguard use among athletes. The most common reasons for not using a mouthguard included the annoying feeling they caused (n = 43, 44.3%), lack of knowledge about the preventive role of mouthguards against TDIs (n = 18, 18.6%), and believing in the ineffective of mouthguards (n = 12, 12.4%) (Fig. 1).

**Table 3:** Participants’ answers to the questions of self-reported preventive practice regarding orofacial injuries (n = 295)

	Karate n (%)	Taekwondo n (%)	Total n (%)
*Do you wear a mouthguard during training or match?*			
Yes^a^	53 (81.5)	158 (68.7)	211 (71.5)
No	12 (18.5)	72 (31.3)	84 (28.5)
*What type of mouthguard do you use*^b^			
Custom made	4 (6.2)	7 (3.2)	11 (3.7)
mouth-formed	23 (35.4)	67 (29.1)	90 (30.5)
Stock	29 (44.6)	8 (38.3)	117 (39.7)
*Do you lend your mouthguard?*			
Yes	9 (13.8)	4 (1.7)	13 (14.4)
No^a^	46 (70.8)	163 (70.9)	209 (70.8)
*When do you wear your mouthguard?*			
Training only	4 (6.2)	3 (1.3)	7 (2.4)
Matches only	37 (56.9)	91 (39.6)	128 (43.4)
Training and matches^a^	9 (13.8)	79 (34.3)	88 (29.8)
Only when parents or coach tell me	5 (7.7)	11 (4.8)	16 (5.4)
*Do you wear protective headgear?*			
Yes^a^	11 (16.9)	191 (83.0)	202 (68.5)
No	54 (83.1)	39 (17.0)	93 (31.5)

^a^Favorable answers

^b^In this question the answers had weighted scores (custom-made = 3, mouth-formed = 2, stock = 1); there were some respondents who reported no usage of mouthguard

The mean score of self-reported preventive practice was 3.53 ± 1.82 (range: 0–7). The question: “Do you wear a mouthguard during training or a match?” was answered favorably by 71.5% (n = 211) of the respondents and the question: “Do you lend your mouthguard?” was answered favorably by 70.8% (n = 209) of athletes; these two questions had the highest number of favorable answers. Conversely, choosing “What type of mouthguard do you use?” by 3.7% (n = 11) was the least favorable answer (custom-made mouthguard) (Table 3).

The mean score of the knowledge of emergency management of orofacial injuries was 1.67 ± 1.10 (range: 0–5). The most common correct answer was for the question: “What will you do if your tooth becomes loose due to trauma?” (n = 204, 69.2%), and the least common correct answers were for the question: “What will you do in case of tooth avulsion due to trauma?” (n = 15, 5.1%) and “In case of tooth avulsion, how much time do you think you have to put it back in?” (n = 15, 5.1%) (Table 4).

**Table 4:** Participants’ answers to the questions on knowledge of emergency management of orofacial injuries (n = 295)

	Karate	Taekwondo	Total
*What will you do if a part of your tooth is broken and you find the broken part?*			
I will come to dentist with broken piece of my tooth^a^	42 (64.6)	105 (45.7)	147 (49.8)
I will just go to dentist without broken piece	10 (15.4)	26 (11.3)	36 (12.2)
I do not know	13 (20.0)	99 (43.0)	112 (38.0)
*In previous situation, the broken piece of tooth should be kept in which of the followings?*			
Paper tissue	36 (55.4)	73 (31.7)	109 (36.9)
Water/milk^a^	14 (21.5)	45 (19.6)	59 (20.0)
Dry container	1 (1.5)	9 (3.9)	10 (3.4)
I don't know	11 (16.9)	96 (41.7)	107 (36.3)
*What will you do if your tooth become loose by hit?*			
I will go to dentist at same day^a^	42 (64.6)	162 (70.4)	204 (69.2)
It does not need to examine by dentist	8 (12.3)	24 (1.4)	32 (10.8)
I don't know	15 (23.1)	44 (19.1)	59 (20.0)
*What will you do if your tooth is completely knocked out by hit?*			
I will find the tooth, wash it with soap, and go to dentist	10 (15.4)	26 (11.3)	36 (12.2)
I will find the tooth, put it in its place in my mouth, and go to dentist^a^	3 (14.6)	12 (5.2)	15 (5.1)
I will not look for the tooth and go to dentist quickly	17 (26.2)	24 (10.4)	41 (13.9)
I will find the tooth, put in in a napkin, and go to dentist	26 (40.0)	115 (50.0)	141 (47.8)
I do not know	9 (13.8)	53 (23.0)	62 (21.0)
*What will you do if your tooth completely knocked out and become dirty?*			
I will wash it with toothbrush slowly	19 (29.2)	65 (28.3)	84 (28.5)
I will wash it with water^a^	15 (23.1)	52 (22.6)	67 (22.7)
I will put the tooth back in mouth without any manipulation	3 (4.6)	5 (2.2)	8 (2.7)
I will wash it with soap	14 (21.5)	21 (9.1)	35 (11.9)
I do not know	14 (21.5)	87 (37.8)	101 (34.2)
*In case of a knocked out tooth, how much time do you think you have to put it back in?*			
15 min	8 (12.3)	19 (8.3)	27 (9.2)
30 min^a^	3 (4.6)	12 (5.2)	15 (5.1)
1 h	3 (4.6)	9 (3.9)	12 (4.1)
Only a dentist can put it back	51 (78.5)	190 (82.6)	241 (81.7)
*If a tooth gets knocked out what would you put it in?*			
Water	17 (26.2)	30 (13)	47 (15.9)
Milk^a^	5 (7.7)	41 (17.8)	46 (15.6)
Paper tissue	36 (55.4)	105 (45.7)	141 (47.8)
Sports drink	1 (1.5)	0 (0.0)	1 (0.3)
Nothing	5 (7.7)	25 (10.9)	30 (10.2)
I do not know	1 (1.5)	29 (12.6)	30 (10.2)

^a^Correct answers

The score of self-reported preventive practice had a significant relationship with age (β = 0.19, *p* = 0.002) and self-reported experience of orofacial injuries (β = − 0.15, *p* = 0.010). Moreover, gender (β = 0.11, *p* = 0.048) and exercise time per week (β = 0.15, *p* = 0.012) had significant relationships with the knowledge score; female athletes and athletes who exercised more per week had more knowledge score on management of orofacial injuries (Table 5).

**Table 5:** Results of linear regression model controlling for demographics on factor associated with athletes’ self-reported preventive practice and knowledge regarding orofacial injuries (n = 295)

	Unstandardized coefficients	Standardized coefficients^a^	*p* value
*Self-reported preventive practice*			
Age	0.2	0.2	0.002
Self-reported experience of orofacial injuries	− 0.6	− 0.2	0.010
*Knowledge*			
Gender	0.3	0.1	0.048
Exercise time per week	0.2	0.2	0.012

^a^Scale-standardized

The Pearson correlation coefficient showed that self-reported practice had a weak correlation with knowledge (r = 0.16, *p* = 0.006).

## Discussion

The present study was conducted to evaluate self-reported experience of orofacial injuries, self-reported preventive practice, and knowledge of emergency management of sports-related orofacial injuries in adolescent martial arts athletes (Karate and Taekwondo). The results showed that self-reported preventive practice of athletes was moderate while their knowledge of management of orofacial injuries was inadequate. Moreover, the prevalence of previous orofacial injury was relatively high with no difference between males and females. Furthermore, older athletes, athletes with fewer training sessions per week, and those with previous training on prevention and management of orofacial injuries had less experience of orofacial injuries.

In the present study, 62% of the participants reported the history of at least one orofacial injury, which was rather high in young Iranian athletes. This finding was similar to a study by Bolhius et al*.* (62–68%) who evaluated orofacial injuries in field hockey players [26]. However, some studies have found lower rates of orofacial injuries; for instances, about 20% of 8–26 year-old Croatian Taekwondo athletes [27], 14% of Croatian Taekwondo players [8], 50% of Catalonian [28], 16% of Dutch field hockey players [29], and 49% of Croatian professional handball players [30] had experienced orofacial injuries. Different contact sports may pose different risks of oral and dental injuries; thus, based on the risk of orofacial injuries, Federation Dentaire International has categorized sports into high- and medium-risk sports. High-risk sports include American football, hockey, ice hockey, lacrosse, martial arts, rugby, ice skating, skateboarding and mountain biking. Medium-risk sports include basketball, soccer, handball, diving, squash, gymnastics, parachuting, and water polo [3]. Moreover, the occurrence of sports-related orofacial injuries depends on various factors such as the type of the sport, geographical location, age, sample size, level of competition, rules on safety equipment usage, and exposure time [3, 31, 32]. Whilst these differences may be explained by cultural and age diversities within various groups, the methodology of the studies would likely have a significant impact as well.

The present study found no significant difference in the history of orofacial injuries between males and females, which was similar to the findings of Vidovic et al*.* in Croatian Taekwondo athletes [27], and Zaleckiene et al*.* in 11–13 year-old Lithuanian schoolchildren [32]. Some previous studies showed that males experienced more trauma compared to females [33–35]. It has been suggested that males tend to take part in contact sports and violent behaviors more than females do [2, 19]. However, gender differences appear to be fading as more females are participating in sports including contact sports [19, 20]. Traebert et al*.* suggested that females could be exposed to the same risk factors for orofacial injuries as men [20].

The results showed that younger players experienced orofacial injuries more frequently compared to older players. Different studies have shown that the majority of orofacial injuries occur in the age group 9–12 years [2, 19]. Moreover, it has been found that amateur athletes suffer from orofacial injuries more often compared to professionals [3, 16]. The most likely reasons may be lack of professional athletic skills in amateurs and improper use of mouthguards compared to professional athletes. Besides, the more hours of training per week in our study was associated with more self-reported experience of orofacial injuries. Furthermore, the results showed that previous training on prevention and management of orofacial injuries was associated with reduced history of orofacial injuries. Even a short-term training course for non-professional population may improve their knowledge [18]; therefore, the more knowledge, the better the practice, which can lead to less orofacial injury.

In the present study, 71.5% of the participants indicated that they usually wore a mouthguard during a match or practice, which was higher than Korean taekwondo athletes (56%) [36], and Saudi taekwondo athletes (56%) [37]. However, Vivovic et al*.* reported mouthguard use by 96% of young Taekwondo athletes [27]. Bergman et al. studied professional handball players and found that regular use of the mouthguard would reduce the chance of dental injury by 5.55 times [30]. Sepet et al*.* found that whilst 41.1% of participants understood the real possibility of orofacial injuries in sports and 55.4% were aware of the protective effect of mouthguards, only 11.2% of the participants reported routine mouthguard use [38]. Same findings by Galic et al*.* showed that although most of the participants were aware of the protective benefits of wearing a mouthguard in reducing sports-related injuries, only 41% of them actually used it [8].

The athletes’ attitude and perceptions about wearing mouthguards and its comfort influences their compliance [9]. In our study, the most common reason for lack of mouthguard use was the annoying feeling it caused, which was similar to other studies [27, 36–38]. This may be because of the type of the mouthguard used: in the present study, only 3.7% of the participants wore custom-made mouthguards while 39.7% of participants wore stock mouthguards and 30.5% used mouth-formed mouthguards. These findings were similar to a study by Vidovic et al*.* that found that only 5.3% of the participants used custom-made mouthguards [27]. Moreover, these findings are consistent with other studies investigating the use of different mouthguard types in other contact sports, such as studies conducted by Biagi et al*.* [39], Emerich et al. [40], and Andrade et al. [41]. In many studies, athletes reported that using stocks or mouth-formed mouthguards caused breathing and speaking problems or jaw and muscle fatigue, or they fell out or became dislodged during use; however, these problems were minimal in custom-made mouthguards because this type fits well with the gums and teeth [21, 27, 28, 30, 36]. Although custom-made mouthguards are relatively expensive, which is a significant barrier against their wide use, the treatment cost and frequency of TDIs are considerable indicating the importance of prevention of TDIs in the best possible way [3]. Therefore, the American Academy of Pediatric Dentistry and the Academy for Sports Dentistry have recommended the use of custom-made mouthguards in all activities with a risk of TDIs [9, 12]. Another barrier against mouthguard use is poor compliance of athletes [9, 42]; however, mouthguard use recommendation by dentists would encourage their use and improve the athletes' compliance [30].

The outcome of orofacial injury treatment depends not only on the knowledge and skills of the dentists but also on the emergency aid available at the site of the injury [3, 19]. Particularly, in case of tooth avulsion as a dental emergency, early and accurate management is necessary to have the most successful outcome [17]. It is therefore important that athletes and coaches have basic knowledge about how to deal with emergency TDIs [3, 19]. In our study, the athletes’ knowledge score was generally low; the poorest answers were related to the management of tooth avulsion as only 5% of respondents were aware of the possibility of replantation of an avulsed tooth, and less than one quarter of them indicated that using water was sufficient to clean the contaminants of an avulsed tooth. Generally, these findings are in accordance with similar studies that showed low knowledge levels of athletes regarding management of TDIs, particularly tooth avulsion [8, 37–39, 43, 44]. This is a worrying finding that requires the dental professionals to interact with the sports community to improve their awareness regarding prevention and management of sports-related orofacial injuries. Dentists could play an important role in educating athletes by demonstrating the benefits of using custom-made mouthguards and encouraging them to use one. Many studies have suggested education as a preventive measure to improve athletes’ knowledge of prevention and emergency management of TDIs [8, 38, 39, 45]; this goal can be easily achieved using different methods such as professional lectures, video materials, and/or text materials such as posters and leaflets.

To the best of our knowledge, this was the first study of the preventive practice of Iranian adolescent athletes regarding orofacial injuries and their awareness about the management of TDIs. The participation of a high number of female adolescence athletes was one of the main strengths of this study. However, this study had some limitations. Similar to other questionnaire-based studies, athletes were required to remember their mouthguard use and previous injuries, which increased the possibility of recall bias and social desirability. Furthermore, the questionnaires did not consider the fact that some individuals might have started using a mouthguard after an orofacial trauma [10]. However, the findings are valuable in developing preventive strategies in sports activities for young athletes.

## Conclusion

The study showed a relatively undesirable preventive practice and distinct lack of knowledge regarding the emergency management of orofacial injuries. The main reason for not using mouthguards was the annoying feeling they caused, probably because most of the athletes used stocks or mouth-formed mouthguards that were unfitted. It seems that provision of education by dentists to all people who interact with young athletes, including parents, teachers, coaches, and the athletes themselves is vital to promote oral health in athletes. Making mouthguard use compulsory in sports with a medium risk of orofacial injuries is as important as that in high-risk sports. The high occurrence of self-reported orofacial injuries indicates the importance of more education and stricter rules for the athlete population.

## Data Availability

Not applicable.

## Abbreviations

TDIs: Traumatic dental injuries
COVID-19: Coronavirus disease of 2019
SPSS: Statistical package for the social science
